# Functional evolution and functional biodiversity: 150 years of *déjà vu* or new physiology of evolution?

**DOI:** 10.3389/fcell.2024.1485089

**Published:** 2024-10-24

**Authors:** Leonid L. Moroz, Daria Y. Romanova

**Affiliations:** ^1^ Department of Neuroscience and McKnight Brain Institute, University of Florida, Gainesville, FL, United States.; ^2^ Whitney Laboratory for Marine Bioscience, University of Florida, Saint Augustine, FL, United States.; ^3^ Institute of Higher Nervous Activity and Neurophysiology of RAS, Laboratory of Cellular Neurobiology of Learning, Moscow, Russia

**Keywords:** evolutionary physiology, evo-devo, Baldwin effect, ecosystems, exaptations, nervous system evolution, phyla, learning and memory

## Introduction

Research at the intersections of different scientific fields is the primary catalyst to solicit novel disciplines and expand the frontiers of knowledge. Quantum physics in chemistry and biology, artificial intelligence, nanoscience, and advances in genomic revolution with cost-efficient sequencing conceptually and radically changed scientific paradigms with profound public and economic impacts. The current Frontiers’ research topic further stresses the need for integrative studies, advocating *Eco-evo-devo* as an emerging discipline of biology by expanding “traditional evo-devo” to ecology.

On a deeper level, this is a recurring and persistent *déjà vu* in the history of biology ever since Darwin-Wallace papers (Darwin and Wallace, 1858), when it was confirmed, and it is still impossible to understand biological phenomena without deciphering evolutionary mechanisms at *all* levels in *changing ecosystems* (using modern terminology). Arguably, this “first rigorous formulation of the concept of evolution, made just over a century ago, was the most useful one we have ever had” (Carneiro, 1972). In his masterpieces, Darwin bridged geology, ecology, biogeography, biodiversity, different types of selection, development, psychology and behaviors (Darwin, 1872; Darwin, 1880). Today, the unification of disciplines for mechanistic and systemic understanding of evolutionary processes reflects the still ongoing scientific and societal challenges under an accelerating information tsunami. Here, we emphasize two aspects of current challenges demanding integrative approaches.

The first problematics is the bottleneck in education, when the evolutionary training and biosystematics courses, which deal with the most fundamental concepts in biology, have quietly lost their place of eminence within the biomedical curriculum—“outcompeted” by escalating specialization and the increasingly technical nature of many disciplines (Moroz, 2010).

**FIGURE 1:**
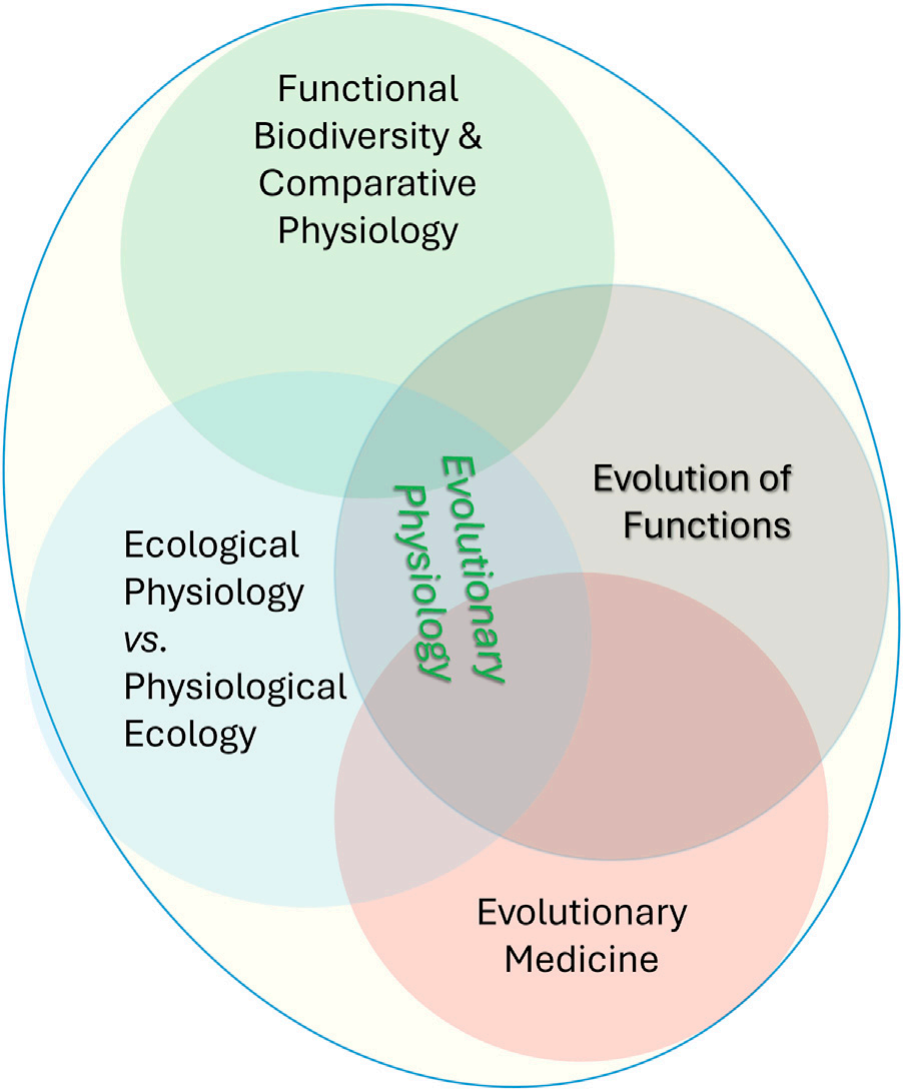
**Functional Evolution** and its subdisciplines were also united as classical Evolutionary Physiology. Functional evolution highlights the importance of understanding how all biological *functions* evolve, from the molecular level to the whole organism, and how these changes contribute to species adaptation, survival, and diversification. Under this framework, the emerging, conceptually similar term **Physiology of Evolution** further illuminates the importance of physiological approaches to understanding evolution. Notable, these terms and research strategy do not revisit the concept of evolution; we emphasize the urgent need for paying more attention to real-time physiological aspects of various biological traits, focusing on integrative mechanisms such as neuronal, hormonal, and immune functional interactions at the top of the organismal and behavioral hierarchies.

The second problematics is an equally critical bottleneck in our understanding of functional evolution (Figure 1) as hierarchical architectures of *real-time* physiological and metabolic/biochemical *interactions* underlying mechanisms of adaptations that can explain the biodiversity dynamics on the changing planet. The incorporation of a physiological web of life into “genomic” evolution will better forecast biota’s resilience to environmental stressors. That is functional biodiversity–the strategy to *marry physiology at all levels of organizations (from genomes to behaviors) with classical biodiversity to understand micro- and macro-evolution*. We stress the more integrative term of *functional evolution* in changing ecosystems rather than the evolution of particular functions, that is, the multilevel reconstruction of the history of individual traits from proteins to behaviors, also known as evolutionary physiology. In these definitions, numerous details of the evolution of diverse functions in cells, tissue, and organs’ systems have to be united as components of functional evolution (Figure 1). In the broadest sense, we should not differentiate the terms “functional evolution” from “evolution” as such. We emphasize that the current evolution studies need significantly more physiological approaches rather than over-dominance of gene-centric approaches by continuing to focus on genes and genomes and then “jump” to organismal phenotypes without real-time physiological studies. Thus, modern *physiology of evolution* is a richer and broader framework, where a web of functions is understood at different levels, as opposed to the gross (organismal) definition of function in changing ecosystems.

**FIGURE 2:**
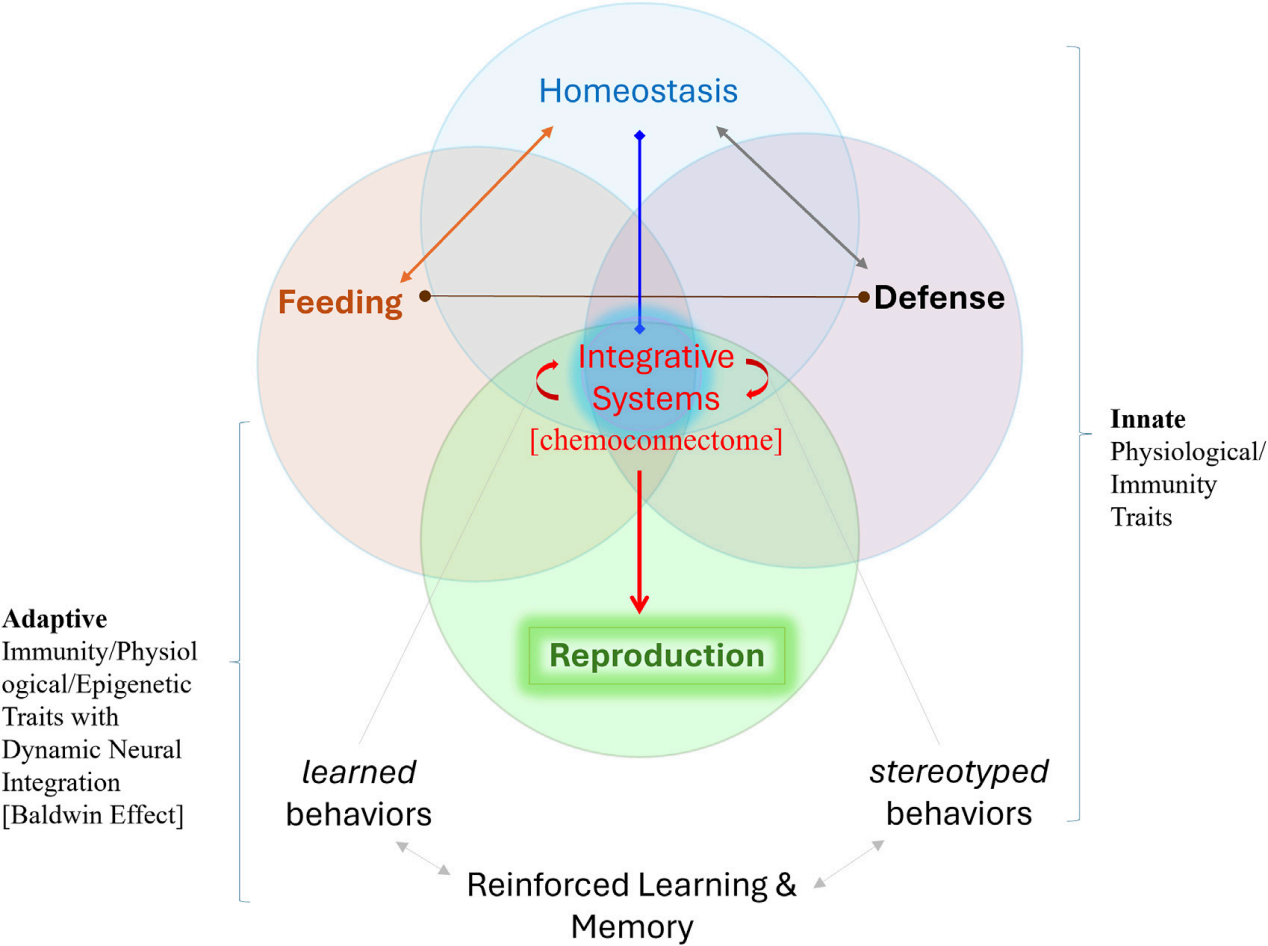
Five Integrative Systems with reproduction at the top of the behavioral hierarchy and widespread neuronal controls. The integrative systems primarily interact using chemical signaling or the chemoconnectome, which embraces a broad spectrum of electrochemical communications with hundreds of (neuro)transmitters and hormones. The chemoconnectome is the core of most ancestral architectures of integrative mechanisms, inherently coupled across all levels of biological organization in evolution. The spectrum of emerging physiological and neurobiological mechanisms in changing ecosystems can dramatically increase the adaptive space for survival, evolutionary innovations [=Baldwin effect-(Baldwin, 1896c; Morgan, 1896; Osborn, 1896; Baldwin, 1897; Loison, 2021)], and reproductive success, often with more progeny (Hinton and Nowlan, 1987; Smith, 1987; Anderson, 1996). Here, we stress that real-time integrative systems are essential for all evolutionary novelties and adaptations, with nervous systems and adaptive behaviors at the top of the organismal hierarchy for most animal lineages. “Integrative activity” occurs at all levels: from molecules and genome operations to organs and whole bodies, emphasizing the fact that hierarchical integrated systems are subjected to selection also at different levels and with emergent properties at each level. This framework unites physiology and ecology approaches to understand evolutionary processes.

Competition and selection occur at all levels of physiological integration, requiring more research on traits critical to decision-making, including learning and memory, homeostatic mechanisms, adaptive metabolism, and immunity (Figure 2). We especially point out the necessity of understanding organisms as highly dynamic hierarchical systems subjected to selection that work on semi-autonomous integrated systems with emergent properties at each level of increased complexity. Such architectures can be viewed as the collective intelligence of heterogeneous cell populations both in simpler and complex metazoans (from placozoans to cuttlefishes) with social behaviors.

Functional evolution also embraces the phenomena of physiological selection (Romanes and Moulton, 1886) and the so-called Baldwin effect (Baldwin, 1896c; Morgan, 1896; Osborn, 1896; Baldwin, 1897; Simpson, 1953; Newman, 2002; Sznajder et al., 2012). Here, the ability to adaptability accelerates evolution by forming, and even making, novel internal and environmental adaptive spaces, therefore giving extra time for natural selection to act; and favoring a broader range of physiological, epigenetic, stereotyped, and learned behavioral outcomes as feedback mechanisms and platforms for functional evolution.

What is remarkable–these critical integrative questions and directions have been recurrently emerging over and over again for 150 years, sometimes with changes in the scope and meaning of terms. Thus, it would be informative to refer to the history of terms briefly, and then summarize past and modern frameworks.

## What is physiology?

Physiology is somewhat a missing elephant in a palace of evolutionary biology today and evo-devo in particular. The term physiology is derived from the ancient Greek φύσις *(phúsis -* “nature, origin”) and -λογία *(-logía -* “study of”) (Fletcher, 1835; Gontier, 2024) and traced back to 1628 - the classical work of William Harvey on blood circulation with a remarkable integrative conceptual framework (Harvey, 1628; Whitteridge, 1964; Whitteridge, 1978). The American Physiological Society defines physiology as the *science of life* (https://www.physoc.org/explore-physiology/what-is-physiology/), and its primarily based on dynamic cellular-molecular interactions. In this sense, the subject of physiology is the *living* systems (i.e., cells and organisms with self-autonomous respective integrative functions) vs. studies of *non-living* systems, which include the deciphering of gene sequences, or locations of their expression, subcellular molecular ensembles (and even gene regulatory networks as an array of regulatory components vs. *functional* networks within defined cell and organismal homeostasis identity and behaviors).

For all definitions, the key to the strategy of physiology is the *integration* and *real-time* interactions of organismal multifunctional networks to obtain energy, survive, and reproduce (Figure 2). In this sense, Darwin and Wallace were comparative integrative physiologists and ecologists who worked to decipher the origin of species. In fact, Darwin was a member of the Physiological Society from the very beginning of its foundation in 1876 (Sharpey-Schafer, 1927), collaborating with physiologists on mechanisms of selection (Romanes and Moulton, 1886), see details in (Noble and Noble, 2023a).

## From classical evolution to functional evolution

Literally, the word evolution means “*unrolled*,*”* which refers to ancient books that were rolled on wooden rods (McCabe, 1921), gradually transforming to Bonnet’s unfolding of the human embryo from a perfectly formed “homunculus,” and eventually to the concept of “descent with modification,” theories and facts (Mayr, 2001) illuminating causes, tempo, and mechanisms of the 3.5+ billion-year life history on our planet with 10–100 million of extant species, including 33 animal phyla, changing global ecosystems.

As noted by (Carneiro, 1972), because of the original doctrine of *preformations* (e.g., reading of an already written text in rolled books or unfolding of a tiny preexisting homunculus), Lamark did not use the term evolution in his famous book (Lamarck, 1809), focusing on the role of physiology in the development of emerging properties. Interestingly, Darwin also did not use the term evolution throughout the text of the first five editions of his “Origins of Species.” Darwin concluded, however, his masterpiece with the word “evolved.” Spencer’s evolution terminology preempted biology (Spencer, 1863) with the famous “survival of the fittest” at the *complex interplay with the environment*.

Soon, evolutionary embryology concepts led to impactful theories of animal origins, known as *Gastrea* (Haeckel, 1874) and *Phagocytella* (Metschnikoff, 1886). Thus, integrative and comparative biology at the end of the XIX century can already be viewed as *eco-evo-devo* in modern terms. Furthermore, Metschnikoff’s phagocytella concept included the unification of *cell physiology* and *immunity* (phagocytosis) to explain system mechanisms of the germ layers’ formation as predecessors and factors for functional evolution in general and the origins of developmental processes (e.g., gastrulation) in particular. These views were conceptually different from past preformation theories by employing dynamic physiological processes (rather than static morphology) as subjects of selection and adaptation.

## Questions of “*how*” and “*why”*

The evolutionary focus shifted early to real-time physiology, where mechanistic questions “*how”* (“what for“) something works were coupled with the questions of “*why”* this system works in such a particular way (or “*how come”* - (Mayr, 1961)) as a result of its genealogy and long planetary history. Why do we observe certain types of cellular or systemic organizations in ctenophores, placozoans, cnidarians, molluscs, arthropods, or chordates instead of others? These “*why*” questions target both primary causes and exaptations (Gould and Vrba, 1982) to reconstruct the extant functional biodiversity in ecosystems.

Why, for example, are individual neurons so different from each other within a given species and across phyla? One possible answer is the functional demands within a given neural circuit and behavior. Another is that each distinct neuronal population forming complex neural nets or brains has a different evolutionary history, and, as a result, neurons carry the heavy molecular burdens of primordial integrative systems within their complex evolutionary past–*neurons are also different because they have different genealogies.* In other words, neurons might independently evolve from distinct types of secretory cells with different secretory products and functional interactions. These multilevel interactions could be preserved over many million years, therefore explaining the astonishing diversity of signal molecules in the brains and complex dynamics of extant chemoconnectomes (Figure 2) as a result of their deep ancestry (Moroz, 2014; 2021). Such distinctive cellular ancestries and integrative mechanisms might either limit or facilitate future evolutionary opportunities to adapt to changeable environments. In other words, past evolutionary history might provide constraints for the emergence of novel behaviors within a given time or resistance to stress, disease, or injury. Remarkable examples of extensive parallel evolution of physiological functions (compared to morphology) exist within all animal phyla, enabling forecasts for resilience (or not) to anthropogenic or climate changes. Yet, new experimental designs are needed to reveal and explain the origins or loss of biological complexity, and past or ongoing extinctions of species and ecosystems.

As stressed by one of the reviewers: “Understanding the “*physiological functional web of life*” in modern terms means gaining better purchase on *how*, *why*, *when*, and *with whom* eukaryotic cells choose to partner for their physiological mechanisms that propel evolutionary solutions to cellular problems.” Holozoan animal ancestors evolved in Ediacaran ecosystems dominated by prokaryotes. And, as in today’s ecosystems, microbiome complexity continues to shape, drive, and even dramatically change paths of evolutionary trajectories via cross-kingdom signaling (Heyland and Moroz, 2005) and horizontal gene transfer (HGT) with omnipresent viromes, further contributing to animal innovations via transposon-derived transcription factors (Mukherjee and Moroz, 2023), for example. By itself, it fully justifies the efforts to preserve the entire biodiversity from microbes (Averill et al., 2022) to all eukaryotes as primary preconditions of Planetary Health (Bertram et al., 2024).

## Past and modern frameworks for evolutionary physiology

Even Aristotle noted that living organisms are causes of themselves (Okasha, 2024), which, in modern terms and our perception, refers to the physiology. As a separate discipline, evolutionary physiology was not established for nearly a century since Darwin (Orbeli, 1941; 1961; Natochin and Chernigovskaya, 1997; Svidersky, 2002; Natochin, 2009; Natochin, 2017). In part, this situation was due to the complexity of processes and emerging systemic properties of multicellular interactions across phyla. The deficit of studies on the evolution of functions was clearly recognized early in the XX century (Lucas, 1909). The term “*evolutionary physiology*” was coined by A.N. Severtzov in 1914 to complement what was already established by that time as evolutionary morphology and development (Severtzov, 1914; Natochin, 2010). Physiology was then and remains today the most integrative approach for functional evolution, inherently focused on real-time interactions of myriads of molecular and cellular processes leading to organismal homeostasis with stereotyped and learned behaviors.

The original framework of evolutionary physiology and functional evolution (Figure 1) included the natural integration of (*a*) comparative and ecological physiology coupled with neural controls, (*b*) ontogenesis, (*c*) clinical studies, including stress, recoveries (or not) from numerous pathologies, and (*d*) the development of unique experimental methodology (Orbeli, 1941; 1961). If the first two approaches were traditional from the very beginning of evolutionary thoughts, the remaining two were entirely novel in the 1940s–1960s and still not yet well established to enable sufficient cross-links across fields. Leon Orbeli developed an earlier strategy for evolutionary physiology (Figure 1), specifically with interpretations of medical pathologies in evolutionary terms as well as insights into the evolution of ion transport and homeostasis (Orbeli, 1941; 1961; Ginetzinsky, 1996). 60 years ago, in the USSR, under the leadership of Orbeli formed the laboratory, and then the Institute of Evolutionary Physiology and Biochemistry (1956); the Journal of Evolutionary Biochemistry and Physiology was established in 1965. A new wave of reviews occurred 30 years later (in the 1990–2000s), calling for a renaissance in the field with more focus on molecular mechanisms (Garland and Carter, 1994; Feder et al., 2000; Garland et al., 2016; Galván et al., 2022).

Today, with many thousands of genomes sequenced, evolutionary approaches in biomedicine have gained momentum with successful stories that explain specific adaptations to various pathogens, preconditions, preventive diagnostics for Mendelian diseases, and forecast of outcomes as parts of personalized medicine. Most pathologies might recruit or be constrained by ancestral gene/cellular regulatory programs and signaling networks under stressful disease-driven tissue microenvironments. For example, particular modern human adaptations or constraints were acquired from the Neanderthal ancestry and beyond, and most of them are deeply embedded in the dynamics of metabolic architectures (Zeberg et al., 2024), rather than specific genes for cognitive capabilities. All these events were affected by countless environmental factors over thousands of generations, with even deeper evolutionary ancestral innovations due to the modularity of multi-domain protein assemblies (McCoy and Fire, 2024; Moroz, 2024).

We highlight the recent strong statements by Denis Noble: (i) *Physiology restores purpose to evolutionary biology* (Noble and Noble, 2023a); (ii) *Genes are not the blueprint for life* (Noble, 2024). In Noble’s words: “The genome is not a code, blueprint or set of instructions. It is a tool orchestrated by the system” (Noble and Noble, 2023b). First, because the vast majority of genes do not have a single “pre-set” function that can be determined from their DNA sequence with perfect examples of multi-modal roles of ion channels in control of cells and organismal behaviors (Noble, 2021) due to the compensatory redundancy of regulatory systems; second, there are emerging, often unpredictable, properties of complex living systems, which can not be deduced from properties of individual components. Here, we emphasize that only real-time physiology in natural habitats (*in situ*) can experimentally unroll the integrative logics of life as we know it, perhaps under the unified theme to proactively “explore” the environment to “learn” to “eat” to reproduce.

Admittedly, we do not know how deep in time we can trace the origins of organismal integrative systems (e.g., neural, hormonal, and immune - (Moroz, 2021)) or mechanisms of genome-scale integration in any particular cell type genealogical lineage. Indeed, cancer is a disease of the genome operation within specific cellular and organismal contexts. Integrative properties of 3D genome operation with thousands of co-expressed genes in each cell are largely unknown and often referred to as a ‘genomic dark matter’ with undetermined redundancy. With trillions of individual cells in the human body, with more than 10,000 differentially expressed genes in each cell type, the task of uncovering such complexity seems to be impossible. Traditional knockout approaches or gains of particular functions are often not sufficient, due to compensatory mechanisms and redundancy of molecular and system signaling and with limited success of a small number of so-called model organisms representing a few specialized animal lineages out of 30+ phyla and 100+ classes of extant metazoans (Moroz, 2018). As a result, a broader concept of ***reference species*** has been introduced (Striedter et al., 2014), stressing the importance of studying diverse taxa from multiple ecological niches across all phyla.

We are confident that the complementary comparative ecophysiological strategy is needed and possible; it can be and should be executed in the nearest time by proactively learning from experiments performed by Mother *Nature* over 3.5 billion years of biological evolution with the advent of floating field laboratories (Moroz, 2015). Pioneering work led by Knut Schmidt-Nielsen (Schmidt-Nielsen, 1997) and George Somero (Somero and Hochachka, 2002; Somero et al., 2017; Somero, 2022) in ***ecophysiology*** of system, cellular and biochemical adaptations validate both feasibility and conceptual breakthroughs in these integrative strategies.

Today, ***4D+ (space and time) single-cell multi-omics and real-time imaging of cellular dynamics*** can be efficiently integrated with the ecophysiology of life cycles, including the identification of functional networks from heterogenous cell populations and signal molecules with deciphering events of convergent evolution and recruitments of homologous cell lineages, therefore ***transforming the animal tree of life into the cell type trees and physiological functional web of life***. However, novel technologies are required for real-time imaging and quantification of molecular and cellular dynamics to probe the interplay between eukaryotes, bacteria, archaea, and viromes in changing ecosystems.

## Conclusion and future directions

“Physiology became completely excluded from evolutionary biology and, in many countries, evolutionary biology was no longer taught within physiology and medical courses in universities. Nor has physiology been taught in Evolutionary Biology courses” (Noble, 2023). Denis Noble calls it “a profound mistake,” associated with a gene-/genome-centric view of evolution. Although ideas of evolutionary physiology were introduced in the XX century, they apparently lost their influence on modern evolutionary theories.

We argue that eco-evo-devo can’t “live and survive” without real-time physiology. *In situ*, comparative real-time physiological studies in natural habitats (not in the lab cages) are urgently needed for functional biodiversity and physiology of evolution at all levels of biological organization, from cells to behaviors. We must focus on currently neglected so-called “minor” phyla such as Placozoa and Ctenophora, Dicyemida and Orthonectida, and 20+ others (Nielsen, 2012; Moroz, 2018), which, by their relative simplicity and phylogenetic position, represent crucial reference species to integrate hundreds and even thousands of individually traced signaling pathways from cells to behaviors.

What are physiological processes that cause and drive “functional” evolution? Here, we view the *behavior as the pacemaker of evolution* (Mayr, 2001), critical for most ecological adaptations and stress resilience on the changing planet. As indicated by one of the reviewers: “*learned behaviors characterize all of evolution from its beginnings*,” placing behavior before genes.

We know little about three fundamentals.

(1) What make and integrate complex hierarchies of stereotyped and learned behaviors at the cellular-molecular level?
(2) How do these behaviors *affect* genes, neuron-specific genomic changes, and functional connectivity across cell types and species?
(3) How do behaviors, elementary and complex cognitions, or learned “intelligence” of multicellular aggregates (Levin, 2023) trigger subsequent genetic flow in populations, potentially reinforcing adaptive behavioral patterns?

The recognition of these physiological processes in evolution, known as the Baldwin factor (Baldwin, 1896c; Morgan, 1896; Osborn, 1896; Baldwin, 1897; Simpson, 1953), has grown with the evidence of its accelerated importance in natural selection (Hinton and Nowlan, 1987; Smith, 1987), adaptability, with enigmatic origins of complex innate behaviors or instincts (Baldwin, 1896b; Baldwin, 1896a; Dennett, 2003; Bateson, 2004; Pigliucci, 2005; Crispo, 2007; Badyaev and Uller, 2009; Sznajder et al., 2012; Loison, 2021).

Nearly all behaviors can be modified by learning. Phylogeny of learning is traced to the dawn of animal evolution as a memory of injury (Walters and Moroz, 2009) mediated by a conservative toolkit of small signal molecules and secretory peptides (= chemoconnectomics (Moroz et al., 2021; Moroz and Romanova, 2023) that expand cellular dynamics and phenotypic plasticity at all levels. Organismal behavioral learning can dramatically increase survival; it occurs in somatic cells (not germ cells or gametes) and then affects genes as tools (Hinton and Nowlan, 1987; Smith, 1987). Pioneering neuroplasticity studies on numerically simpler neural systems of *Aplysia* and kin revealed rapid epigenetic changes by co-opting DNA and RNA methylation machinery and piwi genes as toolkits (Day and Sweatt, 2010; Rajasethupathy et al., 2012; Kandel et al., 2014; Pearce et al., 2017; Bedecarrats et al., 2018; Carney, 2018; Yang et al., 2018; Kim, 2019; Huang et al., 2023). Thus, behaviors, learning, and memory give time and space for evolutionary playgrounds.

The quest for innate or learned integrative mechanisms also inherently reopens the discussion and physicochemical definition of “agency” and “purpose” in evolution (Mayr, 1961;Corning et al., 2023;Noble and Noble, 2023a;b). How would any such Darwinian “agency” (Levin, 2023) integrate cell-cell dynamics and propel physiological interactions and adaptability in general? The modern framework of the biological agency, as a self-autonomous organism (Okasha, 2024) with individuality and apparent “goal-directness,” is also a testable hypothesis with a focus on experimental deciphering emerging properties (from cells to ecosystems) that are not directly forecasting from a gene-centric approach. Here, “organism-as-agent” heuristic experimental motivation can be viewed as not that cell ensembles or organisms “consciously aim to maximize inclusive fitness in their social interactions, but rather they behave as if they do” (Okasha, 2024). It would require an understanding of still elusive integrative logics of life, hopefully over the next century, which is an optimistic forecast.

All species continue to evolve together with their symbionts and parasites in land and ocean ecosystems. Rephrasing Peter Medawar, we conclude the alternative to thinking *in evolutionary physiological* terms is not to think at all. In the conceptual sense, “physiology” means “Logic of Life” (Noble, 2023). The sooner the *physiology of evolution* and *functional biodiversity* are inherent and required parts of *every* biomedical student’s curriculum, the greater progress we can expect from a new generation of scientists in the clinic, the laboratory, and in natural ecosystems. Perhaps we need to include evolution and biodiversity, Darwinian ‘agency’ in the curriculum not only in medical and all biomedical training (evolutionary medicine) but also as a crush course(s) (introductory lectures, principles) in the curriculum of chemists, physicists, bioengineers, and mathematicians/computer/AI scientists worldwide for the Planetary Health and interdisciplinary Frontiers across scientific fields and politics.
